# ASSOCIATIONS BETWEEN COVID-19 VACCINE HESITANCY AND SOCIOSPATIAL FACTORS IN NYC TRANSIT WORKERS 50 YEARS AND OLDER

**DOI:** 10.1093/geroni/igac059.1780

**Published:** 2022-12-20

**Authors:** Gabriella Meltzer, Jordan Harris, Michelle Heffner, Paula Lanternier, Robyn Gershon, Alexis Merdjanoff

**Affiliations:** Columbia University, New York, New York, United States; Purdue University, West Lafayette, Indiana, United States; Colorado State University, Fort Collins, Colorado, United States; University of Texas at Austin, Austin, Texas, United States; New York University School of Global Public Health, New York, New York, United States; New York University School of Global Public Health, New York, New York, United States

## Abstract

This analysis aimed to investigate how age, race/ethnicity, and geographical location contributed to vaccine hesitancy in a sample of New York City (NYC) Metropolitan Transit Authority (MTA) workers. Transport Workers Union, Local 100 members completed online surveys in August 2020 about their COVID-19 history, workplace protections and policies, fear of COVID-19 exposure, vaccination attitudes, and sociodemographic and health characteristics. We conducted univariate and bivariate analyses, followed by multivariate logistic regression, to determine the association between respondent age (younger than 50 vs. 50+) and vaccine hesitancy (willing vs. unwilling/unsure). We also produced spatial visualizations to examine these factors by participants’ zip codes. Of 645 respondents, 59% were 50 years or older, 53% were non-White, and 71% expressed vaccine hesitancy. MTA workers ages 50+ were 46% less likely to be vaccine hesitant than their younger counterparts (OR 0.64; 95% CI 0.42, 0.97). Compared to Whites, non-Whites (OR 3.95; 95% 2.44, 6.39) and those who did not report their race (OR 3.10; 95% CI 1.87, 5.12) were significantly more likely to be vaccine hesitant. Those who were not concerned about contracting COVID-19 in the community had 1.83 greater odds (95% CI 1.12, 2.98) of being vaccine hesitant than those who were concerned. Spatial visualizations revealed that the oldest respondents tended to reside in Queens. Zip codes with high vaccine hesitancy were clustered in Brooklyn, where non-White respondents tended to reside. The trends observed in COVID-19 vaccine hesitancy based on race and age persist in a population of high risk, non-healthcare essential workers.

